# RASSF1A protein expression and correlation with clinicopathological parameters in renal cell carcinoma

**DOI:** 10.1186/1471-2490-8-12

**Published:** 2008-09-26

**Authors:** Hossein Tezval, Axel S Merseburger, Ira Matuschek, Stefan Machtens, Markus A Kuczyk, Jürgen Serth

**Affiliations:** 1 Department of Urology, Medizinische Hochschule Hannover, Hannover, Germany; 2 Department of Urology, Marien-Krankenhaus, Bergisch-Gladbach, Germany

## Abstract

**Background:**

Epigenetic silencing of RAS association family 1A (RASSF1A) tumor suppressor gene occurs in various histological subtypes of renal cell carcinoma (RCC) but RASSF1A protein expression in clear cell RCC as well as a possible correlation with clinicopathological parameters of patients has not been analyzed at yet.

**Methods:**

318 primary clear cell carcinomas were analyzed using tissue microarray analysis and immunohistochemistry. Survival analysis was carried out for 187 patients considering a follow-up period of 2–240 month.

**Results:**

Expression of RASSF1A was found to be significantly decreased in tumoral cells when compared to normal tubular epithelial cells. RASSF1A immunopositivity was significantly associated with pT stage, group stage and histological grade of tumors and showed a tendency for impaired survival in Kaplan-Meier analysis.

**Conclusion:**

While most tumors demonstrate a loss of RASSF1A protein, a subset of tumors was identified to exhibit substantial RASSF1A protein expression and show increased tumor progression. Thus RCC tumorigenesis without depletion of RASSF1A may be associated with an adverse clinical outcome.

## Background

Clear cell renal cell carcinoma (CC-RCC) as the most frequent subtype of RCC has been described to demonstrate loss and/or alteration of chromosome 3p [1,2]. So far, some tumor suppressors and candidates have been identified on 3p, such as *FHIT *at 3p14.2, *VHL at *3p25 and the RAS association domain family 1A gene (*RASSF1A*) at 3p21.3. *RASSF1A *has been detected to undergo promoter hypermethylation and epigenetic silencing in CC-RCC [3-7]. The RASSF1A protein contributes to cell cycle control, stabilization of microtubules, cellular adhesion and motility [8]. Moreover, RASSF1A interact with the pro-apoptotic kinase MTS1 and apoptosis-inducing interferon pathways [9,10]. Depletion of RASSF1A is associated with enhanced mitotic progression, a higher risk for chromosomal defects [11-13] pronounced cellular motility [14] and raised tumor susceptibility in knock-out mice [15].

The loss of RASSF1A function due to epigenetic gene silencing has been detected in various tumor entities, implicating that RASSF1A is involved in the pathogenesis of a wide spectrum of tumors [8]. Hypermethylation and loss of RASSF1A mRNA expression has been shown for CC-RCC in several studies. While some found methylation in tumor tissues [3-5,16-18] others reported significant methylation occurring also in normal tissue [3,6,17,18]. Considering that the detection of methylation occurring in normal tissue together with hypermethylation detected in corresponding tumor tissue might be indicative for an involvement of *RASSF1A *in the early tumorigenesis of CC-RCC, we have recently carried out a study explicitly aiming at the comparison of methylation in paired normal and tumoral tissues [7].

As a result we found considerable methylation in normal tissues that becomes significantly increased in corresponding tumor samples. Therefore these results support the hypothesis that RASSF1A is involved in early tumorigenesis of CC-RCC. Moreover we found that protein expression is substantially reduced in tumor cells and in a subset of normal tubular epithelial cells of histopathologically normal kidney parenchyma. While these data overall demonstrate an inverse relationship of methylation and protein levels in RCC it is not clear yet whether RASSF1A levels or the degree of epigenetic silencing is associated with clinicopathological parameters of RCC patients. So far, controversial results were reported for other tumors such as lung adenocarcinoma or non small cell lung cancer when analyzing a possible association of RASSF1A expression and grade, stage, metastasis or disease specific follow up of patients [19-22].

In this study we analyzed the presence of RASSF1A protein within the primary tumor of clear cell RCCs and benign surrounding peritumoral tissues using immunohistochemistry (IHC) and tissue microarrays (TMA) and statistically evaluated possible associations of RASSF1A protein immunopositivity and clinicopathological parameters of RCC patients.

## Materials and methods

### Patient characteristics and follow up

The present study included 318 patients, who underwent radical nephrectomy between 1981 and 1998. Tissue was obtained from archival routine surgical specimens. The tissue samples were selected by a pathologist and prepared from the primary tumor as well as peritumoral, histologically benign renal parenchyma and arranged on tissue micro arrays (TMA) as described previously [23]. Tumor samples were classified primarily according to UICC 1997 TNM tumor staging system [24]. At the time of the pathological assessment of our specimens the UICC 2002 version was not available. Moreover, the new pathological classification would not influence our results. Survival analysis was carried out for 187 patients with complete follow-up data and pathologically proved clear cell carcinoma of the kidney. The follow up group exhibited a median age of 57.5 years and a mean follow-up period of 83 (0–248) months (table 1). The male-to-female ratio was 1.4 to one. Seventeen patients demonstrated metastasis at the time of diagnosis whereas 170 of patients had a local tumor in the kidney. However**, **eighteen patients without primary metastasis developed metastases in the course of follow up. Thirty of patients without primary metastasis showed recurrence or metastasis in the course of follow-up. At the time of the last follow-up examination, 93 of patients were alive, 67 patients had died from progressive RCC and 27 patients due to other causes.

**Table 1:** Patient characteristics

**Variable**		**All patients n (%)**	**Follow-up n (%)**
**Total**		318 (100)	187 (100)
**pT stage**	T1	9 (3)	7 (4)
	T2	157 (50)	97 (52)
	T3	137 (43)	77 (41)
	T4	15 (4)	6 (3)
**Lymph node**	N0	162 (51)	96 (51)
	N1	34 (11)	17 (9)
	Nx	122 (38)	74 (40)
**Metastasis**	M0	235 (74)	138 (74)
	M1	63 (20)	35 (19)
	Mx	20 (6)	14 (7)
**pTNM stage**	I	10 (3)	8 (4)
	II	141 (44)	88 (47)
	III	100 (31)	56 (30)
	IV	67 (22)	35 (19)
**Tumor grade**	G1	53 (17)	36 (19)
	G2	200 (62)	134 (72)
	G3	25 (8)	10 (5)
	G4	1 (0.3)	7 (4)
	Unknown	39 (12)	0 (0)

### Immunohistochemistry on tissue microarray sections

The paraffin-embedded TMA samples were stained for RASSF1A protein expression as described previously [7]. Evaluation of TMA's was carried out by counting immunopositive and negative cells within the area of each tissue microarray punch and the diameter of each spot on the slides were 1 mm. In tumor tissues only tumor cells were considered while in peritumoral tissue specimens the number of immunopositive and negative proximal and distal tubular cells but not glomerular cells was determined. The RASSF1A labeling index was calculated as the percentage of cells of interest displaying immunopositivity in the analyzed tissue samples (TMA's spots).

### Statistical analysis

Statistical analyses were performed using the SPSS statistical software (Statistical package for social sciences, SPSS, Inc., Chicago, IL). P-values of < 0.05 were considered statistically significant. Spearman R nonparametric correlation analysis was used to determine the association between RASSF1A immunopositivity and clinicopathological parameters. The Wilcoxon-Mann-Whitney-U-test was applied for statistical comparison of RASSF1A expression as observed in distal and proximal tubules of normal kidney tissue. The possible correlation between cytoplasmic expression of RASSF1A and clinicopathological characteristics was determined by the Chi-square test. Survival was evaluated for patients with RCC of the clear cell type using the Kaplan-Meier method and the log-rank test or Cox regression analysis. Cases with cancer-independent deaths were censored at time of death and included in the analysis.

## Results

### Expression of RASSF1A protein

**Figure 1:**
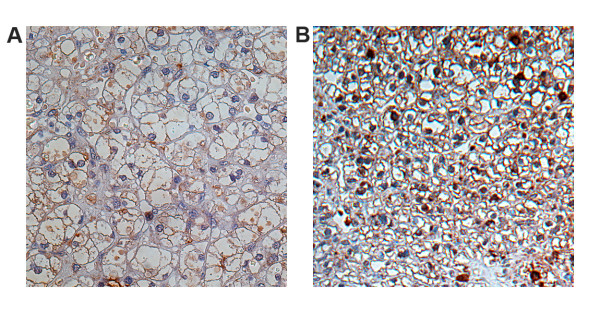
**Differential expression of RASSF1A in clear cell carcinoma of the kidney**. Immunohistochemical analysis of RASSF1A expression in CC-RCC specimens exhibiting < 25% (A) and > 25% (B) labeling index.

**Figure 2:**
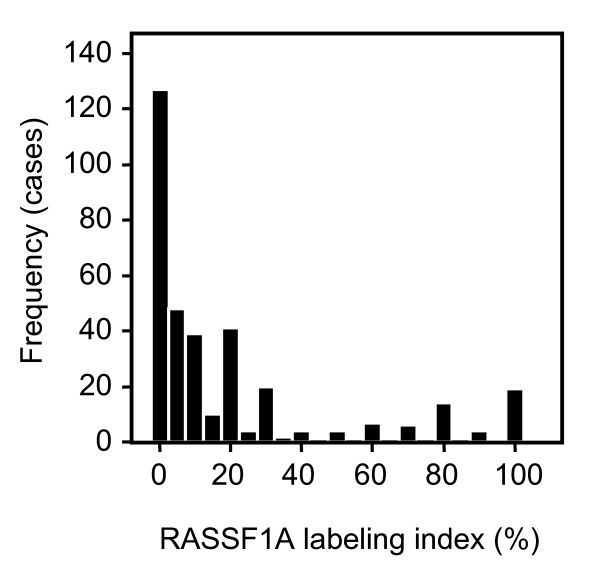
**Frequency plot of RASSF1A immunopositivity in CC-RCC**. Frequency of RASSF1A immunopositivity (**% **labeling index) as observed in clear cell RCC. In approximately 85% of tumors a RASSF1A positivity of less than 25% was observed.

The presence of RASSF1A protein in primary tumor specimens and corresponding histologically normal surrounding parenchyma was investigated using immunohistochemical analysis of TMA slides of 318 patients with CC-RCC. Specific immunopositivity for RASSF1A was exclusively observed in the cytoplasm of tumor and normal tubular epithelial cells [7]. Positivity of RASSF1A was found to be significantly decreased in tumoral cells when compared to normal tubular epithelial cells (p < 0.001, paired t-test). In tumors a mean positivity of 19% (median 11%) was detected and 85% of tumors exhibited a RASSF1A labeling index of ≤ 25% (fig. 1 and 2).

Considering that most of renal cell carcinomas are assumed to derive from epithelial cells of the proximal tubules labeling of RASSF1A protein both in the proximal and distal tubules of histologically normal materials were separately analyzed for a subgroup of 90 tissue samples. An almost complete staining for epithelial cells of the proximal and distal tubules with labeling index of nearly 100% was observed thus no significant difference of RASSF1A immunopositivity between proximal or distal tubules could be found (p = 0.22).

### Association between RASSF1A expression and histopathological parameters

Whether a correlation between immunopositivity of RASSF1A (% labeling index) the primary tumor specimens and the histopathological grading and clinical staging of patients exists, was analyzed using Spearman R nonparametric correlation analysis. In the primary tumor specimens a positive correlation between RASSF1A expression and the pT stage (p = 0.001), histological grading (p = 0.029) and TNM group stage (p = 0.006) was detected, whereas no significant correlation was observed for lymph node metastasis (p = 0.377) and the presence of distant metastases (p = 0.41; table 2). This indicates that higher expression of RASSF1A was positively correlated with the higher pT stage, histological grading, and pTNM group stage in the entire study group.

**Table 2:** Relationship of high RASSF1A labeling index (> 25%) and clinicopathological parameters for the entire study group (n = 318)

Parameter	p-value*
pT-stage	0.001
Lymph node status	0.377
Metastasis	0.410
pTNM stage	0.006
Tumor grade	0.029

*Spearman nonparametric correlation analysis

Taking into account that we recently found a statistical relationship between cyclinB1 expression, as observed in the histopathological normal tissues and clinicopathological parameters [25] we also compared the RASSF1A immunopositivity detected in the paired histopathologically normal specimens to the clinicopathological parameters. No significant correlation between expression of RASSF1A protein in histopathological normal tissue and the parameters pT-stage, lymph node metastasis, distant metastasis, histological grading and TNM group stage of the patients could be detected (data not shown).

### Analysis of RASSF1A protein expression and survival of patients

**Figure 3:**
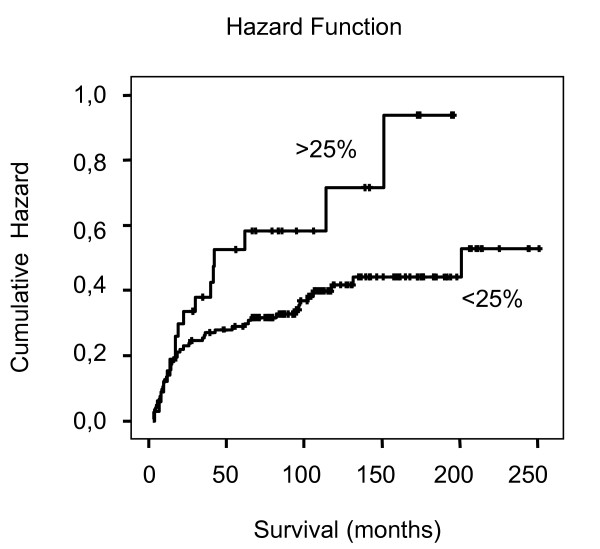
**Kaplan-Meier analysis and Hazard Plot**. Hazard plot illustration of Kaplan-Meier analysis. Disease specific survival of patients demonstrating either low (< 25% immunopositively stained tumor cells) or high immunopositivity (> 25%) is shown. Borderline significance was obtained using log rank test analysis (p = 0.054).

Using a cut-off value of 25% for the relative amount of cells positively stained for RASSF1A in primary tumor specimens, as suggested by the labeling index (fig. 1) to allow discrimination of low and high positivity tumors, a disease-specific survival analysis was performed. Increased positivity of RASSF1A in the tumor samples demonstrated a tendency towards decreased survival in Kaplan-Meier analysis with border line significance (p = 0.054, log rank test), (fig. 3). Using RASSF1A immunopositivity as a continuous variable and Cox regression for survival analysis RASSF1A was not identified as a significant parameter (p = 0.239, hazard ratio = 1.005, 95% CI 0.997–1.013).

## Discussion

RCC is staged conventionally using the TNM staging system. However comparable TNM classifications may demonstrate different clinical courses, indicating the need for additional molecular prognosticators permitting a patient specific staging and future targeted therapeutic treatment.

The role of RASSF1A as a tumor suppressor gene, its silencing and loss of function due to methylation in different tumors as well as in RCC has been reported [3-5,8,26]. Epigenetic inactivation has been observed both in clear cell and papillary RCC [4,6] however, taking into account that 85% of RCCs show clear cell carcinoma histology, only this histological subtype was considered in the present study. To our knowledge this is the first study immunohistochemically investigating a possible relationship of RASSF1A protein level with clinicopathological parameters of patients in RCC. Using tissue microarrays we were able to investigate a large series of specimens of RCC and corresponding histologically normal tissues in a single run thus, minimizing inter assay variances. Our analysis shows that in large part of tumors a loss of RASSF1A positivity is observed. Hence, our data is in concordance with previous reports describing epigenetic silencing of the gene in a substantial proportion of RCCs [4,6]. Therefore, it seems likely that decreased transcription of RASSF1A leads to a loss of RASSF1A protein, such as indicated by our study. Our analysis further supports the hypothesis that RASSF1A could be involved in tumourigenesis of a substantial part of clear cell RCC.

Interestingly, we found that a subset of tumors, showing immunopositivity comparable to that observed in morphologically normal cells, were positively correlated with T, TNM and grade of tumors. Thus, a higher positivity of RASSF1A as observed in this subgroup of tumors is significantly associated with higher stage or grade of tumors but not lymph node or distant metastasis. Moreover, supporting this finding, higher RASSF1A protein levels in tumors demonstrated a possible association with impaired survival of patients.

The clinicopathological significance of alterations of RASSF1A protein levels in tumors has not been described to our knowledge at yet. However, epigenetic silencing associated with a loss of RASSF1A has been statistically analyzed for a relationship with prognostic parameters by a few studies but showed discordant results so far. Thus it has been described that hypermethylation of RASSF1A could be associated with an adverse outcome of patients exhibiting lung cancer [19,20,27,28] whereas other studies found no relationship with prognosis [22,29-31]. Therefore, it is currently not clear whether loss of RASSF1A may serve as a negative molecular prognosticator.

Considering that in our study tumor cells showing normal levels of RASSF1A instead of the most frequently observed loss of positivity, were found to be associated with tumor progression, the question for a potential biological relevance of our findings arises.

Sporadic RCC tumourigenesis has been associated in about 60% of cases with alteration of *VHL *thus preventing ubiquitination and degradation of the transcription factor hyopoxia induced factor 1α (HIF-) even under normoxia in kidney cells [32]. As a consequence deregulation of pivotal cellular processes in tubular epithelial cells of the kidney, such as angiogenesis, cellular growth and acid-base balance has been described to be involved in kidney tumourigenesis. However, it is not known at yet how the presence or absence of RASSF1A in kidney tumor cells interacts with the VHL or other pathways of kidney tumorigenesis.

Loss of RASSF1A expression has been described to be involved in the majority of RCCs and likely plays a role in early RCC tumorigenesis. Thus our protein expression data may give the first evidence that RASSF1A independent RCC carcinogenesis may also occur in a subset of tumors and likely is associated with a relatively declined outcome.

## Conclusion

Loss of RASSF1A protein is observed in the majority of clear cell RCC suggesting a role in RCC tumorigenesis. Depletion of RASSF1A protein is not a mandatory prerequisite of RCC development as a subset of tumors demonstrates protein levels comparable to normal cells. RCC tumorigenesis accompanied by apparently normal levels of RASSF1A is statistically associated with stage and grade of tumors and shows a tendency towards decreased survival. Therefore, it becomes clear that further studies are necessary to specifically characterize the role of RASSF1A in particular with respect to the VHL mediated tumourigenesis in RCC.

## Abbreviations

CC-RCC: Clear cell renal cell carcinoma; *RASSF1A*: RAS association domain family 1A gene; IHC: Immunohistochemistry; TMA: Tissue microarrays; SPSS: Statistical package for social sciences; HIF-1α: Hypoxia ****induced factor 1α; VHL: Von Hippel-Lindau.

## Competing interests

The authors declare that they have no competing interests.

## Authors' contributions

HT carried out the immunohistochemical analyses, statistical evaluation of data and drafting of the manuscript and figures. AM and IM participated in tissue sampling and setup of tissue microarrays and data evaluation. SM and MK participated in the design of the study and performed the analysis of patient data. JS conceived of the study, participated in its design, statistical analysis and coordination and wrote the original and final versions of the manuscript. All authors read and approved the final manuscript.

## Pre-publication history

The pre-publication history for this paper can be accessed here:


